# A Novel Multiscale Gaussian-Matched Filter Using Neural Networks for the Segmentation of X-Ray Coronary Angiograms

**DOI:** 10.1155/2018/5812059

**Published:** 2018-04-18

**Authors:** Ivan Cruz-Aceves, Fernando Cervantes-Sanchez, Maria Susana Avila-Garcia

**Affiliations:** ^1^CONACYT - Centro de Investigación en Matemáticas (CIMAT), A.C., Jalisco S/N, Col. Valenciana, 36000 Guanajuato, GTO, Mexico; ^2^Centro de Investigación en Matemáticas (CIMAT), A.C., Jalisco S/N, Col. Valenciana, 36000 Guanajuato, GTO, Mexico; ^3^Departamento de Estudios Multidisciplinarios, División de Ingenierías, Universidad de Guanajuato, Av. Universidad S/N, Col. Yacatitas, 38944 Yuriria, GTO, Mexico

## Abstract

The accurate and efficient segmentation of coronary arteries in X-ray angiograms represents an essential task for computer-aided diagnosis. This paper presents a new multiscale Gaussian-matched filter (MGMF) based on artificial neural networks. The proposed method consists of two different stages. In the first stage, MGMF is used for detecting vessel-like structures while reducing image noise. The results of MGMF are compared with those obtained using six GMF-based detection methods in terms of the area (*A*_z_) under the receiver operating characteristic (ROC) curve. In the second stage, ten thresholding methods of the state of the art are compared in order to classify the magnitude of the multiscale Gaussian response into vessel and nonvessel pixels, respectively. The accuracy measure is used to analyze the segmentation methods, by comparing the results with a set of 100 X-ray coronary angiograms, which were outlined by a specialist to form the ground truth. Finally, the proposed method is compared with seven state-of-the-art vessel segmentation methods. The vessel detection results using the proposed MGMF method achieved an *A*_z_ = 0.9357 with a training set of 50 angiograms and *A*_z_ = 0.9362 with the test set of 50 images. In addition, the segmentation results using the intraclass variance thresholding method provided a segmentation accuracy of 0.9568 with the test set of coronary angiograms.

## 1. Introduction

Coronary angiography is the standard X-ray imaging procedure used by cardiologists in diagnosing and monitoring vascular abnormalities. In recent years, the development of computational methods to perform image analysis along with computer-aided diagnosis (CAD) has begun to attract more attention. Automatic segmentation of coronary arteries is the main image processing step in cardiology CAD systems and is also a challenging and complex task. The main disadvantages in X-ray angiograms are the uneven illumination and weak contrast between coronary arteries and image background. Given that these two disadvantages generate multimodal histograms, the segmentation task has been commonly addressed in two stages: vessel enhancement also called detection and binary classification also known as segmentation. The first stage is performed to enhance vessel-like structures from the image background while removing image noise, and the second stage focuses on using a soft classification method to differentiate vessel and nonvessel pixels.

Since the automatic coronary artery segmentation stage is an essential task for a number of CAD systems, different computational methods have been introduced for this purpose. In literature, several techniques for working in the spatial image domain with diverse types of vessel detection strategies have been reported. The most basic strategy is based on mathematical morphology [1–5], where the top-hat operator represents the main idea of these methods, obtaining a low performance to detect small vessels. Another types of vessel detection methods are based on the eigenvalues of the Hessian matrix to compute a vesselness measure [6–11]. However, because the Hessian matrix is based on the second-order derivative of a Gaussian kernel, the detection performance can be highly sensitive to noise.

On the other hand, based on the idea of a Gaussian kernel, the Gaussian-matched filters (GMF) [12] were introduced and have been successfully applied in different problems such as image registration [13, 14], retinal vessel detection [15–17], and coronary artery detection [18, 19]. The GMF method is based on a Gaussian template matching used for the detection of vessel-like structures at different orientations. GMF works on the assumption that by using a Gaussian curve, the shape of vessel-like structures can be approximated. To form the Gaussian template, four different parameters have to be tuned. The main parameter is the continuous *σ* value that represents the spread of the intensity profile. The remaining three parameters are defined in the discrete domain. The parameter *L* is the length of the vessel segment to be processed, *T* is the position where the Gaussian curve trails will cut, and *κ* represents the number of orientations in the directional filter bank.

The detection performance of the GMF is directly related to the appropriate selection of the parameter values. Chaudhuri et al. [12] establish the following parameters: *σ* = 2.0, *T* = 13, *L* = 9, and *κ* = 12. Kang et al. [18–21] modified the number of oriented filters (*κ* = 6) along with the *σ* parameter to adapt the GMF to the coronary artery detection problem. Cinsdikici and Aydin [16] increased the number of oriented filters *κ* = 18. To avoid empirical values, Al-Rawi et al. [15, 22] proposed a search space to establish a training step by using an exhaustive search and genetic algorithms, respectively. The search space was used to set the *L*, *T*, and *σ* parameters in the retinal vessel detection problem. Cruz et al. [23] introduced a comparative analysis of four nature-inspired algorithms to obtain the optimal set of parameters of the GMF, to be applied in automatic detection of coronary arteries.

In general, the state-of-the-art GMF-based methods mentioned above assume that by using the average width of blood vessels to form a single-scale GMF, all the vessels in the input image can be detected. To overcome this disadvantage, in the present work, a novel multiscale Gaussian-matched filter (MGMF) based on artificial neural networks is introduced. The proposed method consists of detection and segmentation steps of coronary arteries in X-ray angiograms. In the detection step, MGMF is used to improve the contrast of blood vessels, and it is compared with six GMF-based methods in terms of the area (*A*_z_) under the ROC curve. In the segmentation step, a comparative analysis of ten thresholding methods of the state of the art is performed using the multiscale Gaussian filter response. Finally, the segmentation results of the proposed method are compared and discussed with those obtained using seven specialized vessel segmentation methods in terms of the accuracy measure.

The remainder of this paper is organized as follows. In Section 2, the fundamentals of the Gaussian-matched filters, artificial neural networks, and the proposed MGMF method are described in detail. The experimental results are presented and discussed in Section 3, and conclusions are given in Section 4.

## 2. Methods

Given the suitable performance of the Gaussian-matched filters for detecting coronary arteries in X-ray angiograms, a new multiscale Gaussian-matched filter based on a multilayer neural network is proposed in the present work; this method is described in detail in the present section.

### 2.1. Gaussian-Matched Filters

The Gaussian-matched filters (GMF) were originally proposed by Chaudhuri et al. [12] for detecting vessel-like structures in medical imaging. The main idea behind the GMF is to approximate the shape of vessel-like structures in the spatial image domain by applying a Gaussian template. This template is formed by a Gaussian curve, which can be defined as follows:

(1) $$\begin{array}{c} G\left(x,y\right)=-\exp\left(-\frac{x^{2}+y^{2}}{2\sigma^{2}}\right),\left|y\right|\leq \frac{L}{2}, \end{array}$$

where the variable *L* is used to set the length in pixels of the vessel segment to be detected, and *σ* represents the average width of the vessel-like structures. To establish the width in pixels of the matching template, a discrete parameter *T* must be introduced to define the position where the Gaussian curve trails will cut.

Since the vessel-like structures can appear at different orientations, the Gaussian kernel *G*(*x*, *y*) can be also rotated by using a geometric transformation at different angles *θ* as follows:

(2) $$\begin{array}{c} \kappa =\left[\begin{array}{ll} \cos\theta_{i} & -\sin\theta_{i}\\ \sin\theta_{i} & \cos\theta_{i} \end{array}\right], \end{array}$$

where *κ* represents the number of evenly spaced directional filters *κ* = 180/*θ* in the range [−*π*/2, (*π*/2)]. To obtain the Gaussian filter response, these oriented kernels are convolved with the input image, and the pixels with maximum response over all orientations are preserved.

**Figure 1:**
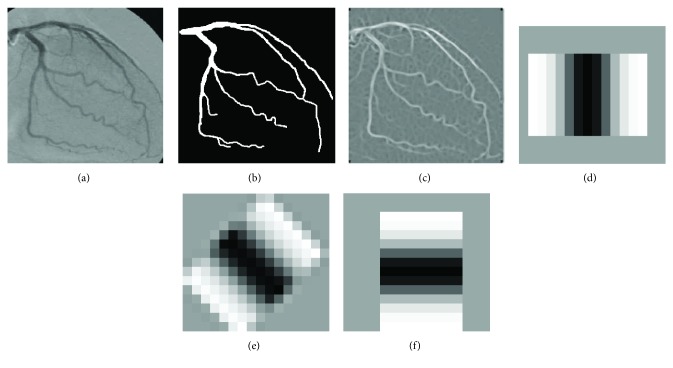
(a) Original X-ray coronary angiogram. (b) Ground truth of angiogram in (a). (c) Gaussian filter response applying 12 directional kernels on the angiogram in (a). (d), (e), (f) Gaussian templates with *θ* = 0°, *θ* = 45°, and *θ* = 90°, respectively, as proposed by Chaudhuri et al. [12].

On the other hand, a tuning step for the four GMF parameters plays an essential role for each application. In Figure 1, an X-ray angiogram along with the ground truth image outlined by a specialist is illustrated.  Figure 1(c) presents the Gaussian filter response obtained using the parameter values proposed by Chaudhuri et al. [12] (*σ* = 2.0, *L* = 9, *T* = 13, and *κ* = 12). Figures 1(d)–1(f) present the Gaussian matching templates with the aforementioned values and with *θ* = 0°, *θ* = 45°, and *θ* = 90°, respectively.

### 2.2. Artificial Neural Networks

Artificial neural networks (ANN) are machine learning techniques inspired by neuron connections in the brain and they are commonly used for classification problems. ANN consists of multiple computing units that resemble to biological neurons connected in a network capable of approximating unknown functions [24]. This network consists of multiple computing units, also called artificial neurons, which perform the weighted sum of their corresponding inputs to be evaluated into an activation function [25]. For each artificial neuron, the evaluation of the activation function is passed as an input for following computing units in the network. This computing units can be arranged in layers that receive the same inputs but use different weights. For a single layer, the weights can be arranged into a matrix *W* of size *n* × *m*, where *n* is the number of neurons and *m* is the number of inputs in the current layer. The computation of one ANN layer can be seen as the evaluation of a matrix-vector product in the continuous activation function *g*(·) as follows:

(3) $$\begin{array}{c} F\left(x\right)=g\left(W^{T}x\right), \end{array}$$

where *W* is the matrix of weight values and *x* is the input vector.

In classification problems, a threshold value for the activation function can be set in order to differentiate between classes. However, the continuous evaluation of the activation function can be used as an universal function approximator.

The performance of the ANN depends on the architecture of the network defined by the number of layers. Each layer contains a number of neurons which are defined by an activation function and an associated weights vector. Commonly, the weights are fitted through a training process while the architecture and the activation functions remain unchanged. For the training of the network weights, the back-propagation method with gradient descent has been widely employed [26]; however, other optimization schemes inside the back-propagation step such as Levenberg–Marquardt algorithm have proved to be more efficient [27].

**Figure 2:**
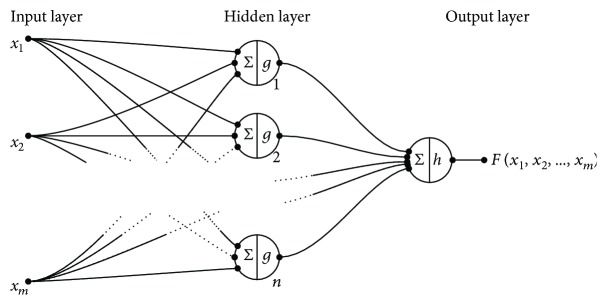
Representation of an artificial neural network with one layer of *m* input values, a hidden layer with activation function *g*(·) and *n* neurons, and an output layer with one neuron and activation function *h*(·).

Figure 2 illustrates the architecture of an ANN with three different layers. The first layer, also called input layer, consists of *m* input values *x* = [*x*_1_, *x*_2_,…, *x*_*m*_], where the input information directly depends on the problem to be solved. In the diagram, the second layer is conformed by *n* neurons: [1, 2,…, *n*] with activation function *g*(·), and it is called a hidden layer because is not relevant to know the result of the function *g*(·) for each neuron. The third layer contains one neuron and has an activation function *h*(·), and it is called the output layer because it returns the evaluation value of the input *x* in the function *F*(·).

### 2.3. Proposed Multiscale Gaussian-Matched Filters

To overcome the main disadvantage of the single-scale GMF-based methods in detecting vessels of different calibers, the proposed method takes advantage of Gaussian curves at different scales in a predefined range *σ* = {1,…, *n*}.

**Figure 3:**
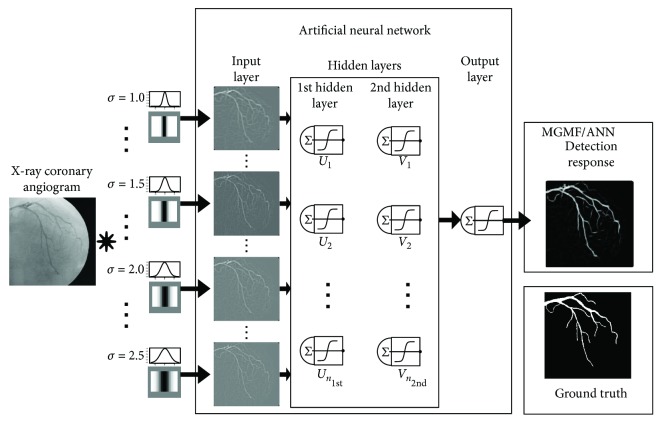
Flowchart of the proposed MGMF vessel detection method.

The flowchart of the proposed method is illustrated in Figure 3. As it can be observed, the procedure of the proposed MGMF vessel detection method consists of four different steps. Firstly, a number of Gaussian scales {*σ*_*i*_ | *i* = 1, 2,…, *n*} must be defined to form a set of Gaussian matching templates. The set of Gaussian scales was determined by using a global search on the training set of images in terms of the area under the ROC curve. In the second step, the Gaussian templates at different values of the *σ* parameter are generated in order to be convolved with the X-ray angiogram input images. Each template is formed according to the *T* and *L* parameters and rotated using the number of directional filters *κ* with angular resolution *θ*. Since the ANNs represent a supervised machine learning technique, in the third step, the resulting Gaussian filter responses and the ground truth images can be arranged as an input data matrix of *n* columns and label vector, respectively. Finally, in the last step of the proposed method, the ANN is trained by a predefined number of hidden layers (the ANN architecture is discussed in Section 3.1). From the ANN, the resulting image represents the vessel detection response, which can be evaluated using a ground truth image and a metric for binary classification.

In the present work, the area (*A*_z_) under the ROC curve is used to select the most suitable set of parameters for the ANN as well as to assess their performance in vessel detection using the training set of angiograms. This measure is explained in the following Section 2.4.

### 2.4. Evaluation Metrics

To assess the performance of the vessel enhancement and vessel segmentation methods, the area (*A*_z_) under the receiver operating characteristic (ROC) curve for gray-scale images and the accuracy metric for binary images have been adopted in this work.

Both evaluation measures are in the range [0, 1], where the value 1 is acquired when the vessel pixels and background image obtained from the computational experiments are completely superimposed with the ground truth provided by the specialist; otherwise, the obtained value corresponds to 0.

The ROC curve is a measure that evaluates the performance of a classification method. This measure is a plot of the true-positive fraction (TPF) also called sensitivity and the false-positive fraction (FPF). TPF represents the rate of correctly classified pixels (vessel pixels) and FPF represents the rate of nonvessel pixels incorrectly classified by the computational method. To compute the ROC curve, a sliding threshold over the gray-scale filter response is computed, and the area *A*_z_ under the curve is calculated through the Riemann sum method.

The accuracy measure [28] has been widely used to evaluate the performance of binary classifiers; consequently, it has been adopted to assess the performance of the vessel segmentation results (binary images). This measure is defined as the rate of correctly classified pixels regarding the number of pixels in the input image as follows:

(4) $$\begin{array}{c} Accuracy=\frac{TP+TN}{TP+FP+TN+FN}, \end{array}$$

where TP and TN represent the subsets of correctly classified vessel and nonvessel pixels, respectively, and FN and FP represent the subsets of incorrectly classified pixels.

In Section 3, the vessel segmentation results obtained from the proposed MGMF method using a database of 100 X-ray angiographic images are analyzed by the evaluation metrics.

## 3. Results and Discussion

In this section, the vessel enhancement and segmentation results obtained from the proposed MGMF method are presented and analyzed. The computational experiments of the MGMF method based on an artificial neural network were implemented on a computer with an Intel Core i3, 4 GB of RAM, and a 2.13 GHz processor using the Matlab software version 2016a.

The database of gray-scale images used in the present work consists of 100 X-ray coronary angiograms of size 300 × 300 pixels from different patients. Each angiogram was outlined by a specialist to form the ground truth images for evaluation purposes. An ethics approval letter was provided by the Mexican Social Security Institute, UMAE Leon. In the experiments, the whole set of X-ray angiograms was divided into the training and testing sets with 50 images in each one in order to assess the vessel enhancement and segmentation methods.

### 3.1. Optimization of the ANN Architecture

A four layered ANN is proposed to detect coronary arteries using the multiscale Gaussian filter response of the X-ray coronary angiograms. The first layer is the input layer, which receives the responses of the GMF at 10 different *σ* values. The second and third layers are hidden layers, which respective number of neurons is defined through an optimization process, described later on this section. The fourth layer is the output layer, responsible for generating the MGMF response.

**Figure 4:**
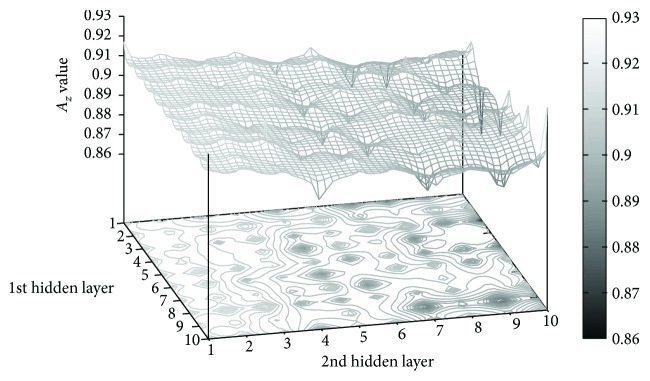
Search space to obtain the optimal architecture of the MGMF, including the number of neurons for each hidden layer of the ANN and using the area *A*_z_ under the ROC curve in the training set of angiograms.

Considering that the performance of the proposed ANN rely on the two hidden layers, an exhaustive search was used to define the number of neurons inside each of them. The search was performed within the space: *n*_1st_, *n*_2nd_ ∈ [1, 2,…, 10], where *n*_1st_ and *n*_2nd_ are the number of neurons inside the first and second hidden layers, respectively. The objective of the exhaustive search was to design the optimal ANN architecture, which must maximize the area under the ROC curve (*A*_z_) using the multiple responses of the MGMF in the training set of angiograms. The parameters of the MGMF were assigned as *l* = 13 and *T* = 15, using *K* = 12 kernel orientations. Those parameters were defined according to the design of the GMF obtained by Cruz et al. [23], using a nature-inspired algorithm to optimize the *l*, *T*, and *σ* parameters, which reported a high detection performance. The average width of the vessels *σ* on the proposed MGMF is defined in the range: [1.5,1.6,…, 2.5], giving a multiscale approach to the detection method. The search space of the *A*_z_ values with respect to the number of neurons inside the two hidden layers is illustrated in Figure 4.

From the exhaustive search results, the optimal ANN architecture was designed to use 3 neurons in the first hidden layer and 8 in the second hidden layer. This optimal architecture will be referred as ANN(3-8) in the remaining of this article.  Table 1 presents a statistical analysis of the proposed MGMF/ANN(3-8) behaviour in the training set of coronary angiograms. The statistical results show a high robustness of the MGMF/ANN(3-8) method according to the low standard deviation of its performance after 30 runs.

### 3.2. Results of Vessel Enhancement

Table 2 presents a comparative analysis between the resulting performance of the proposed MGMF/ANN(3-8) method and six GMF-based methods from the state of the art. In this analysis, the whole set of coronary angiograms was used. The analysis was performed using 7 methods. First, the proposal by Chaudhuri et al. [12] was that the authors defined the GMF parameters experimentally as *l* = 9, *σ* = 2.0, and *T* = 13, with *κ* = 12 orientations. The approach of Cinsdikici and Aydin [16] uses the same set of parameters as proposed by Chaudhuri et al. [12], but changes the number of kernel orientations to *κ* = 18 in order to increase the range of directions of the GMF response. The method of Kang et al. [18, 20, 21] defines the GMF parameters as *l* = 9, *T* = 13, and  *κ* = 6 and modifies *σ* to 1.5 according to the experiments performed by the authors. The approach of Al-Rawi et al. [15, 22] proposes the GMF parameters optimization through a full search and later through a genetic algorithm. The search space defined by Al-Rawi et al. [15, 22] was established as *l* = [7,7.1,…, 11], *T* = [2,2.25,…, 10], *σ* = [1.5,1.6,…, 3], and keeping *κ* = 12 orientations. The method of GMF-Evol [23] compared four algorithms from the evolutionary computation family and defined the parameters for the GMF as the optimal set obtained by the differential evolution algorithm within the search space of *l* = [8, 9,…, 15], *T* = [8, 9,…, 15], *σ* = [1,1.01,…, 5], and keeping *κ* = 12 kernel orientations. Finally, the method GMF-Entropy [19] replaced the area (*A*_z_) under the ROC curve with a nature-inspired optimization algorithm by using an entropy-based objective function for the GMF parameter optimization.

According to the comparative analysis of the computational experiments, the method GMF-Entropy [19] presented the lowest detection performance in both image sets. Also, it can be noticed that the methods of Kang et al. [18, 20, 21], Chaudhuri et al. [12], and Cinsdikici and Aydin [16] that defined the GMF parameters experimentally presented a similar behaviour. The methods of Al-Rawi et al. [15, 22] and GMF-Evol [23] that searched the optimal parameters for the GMF show a slight increment on the *A*_z_ response. Moreover, the proposed method achieved the best detection with an *A*_z_ rate of 0.9357 in the training set and 0.9362 in the testing set of coronary angiograms.

**Figure 5:**
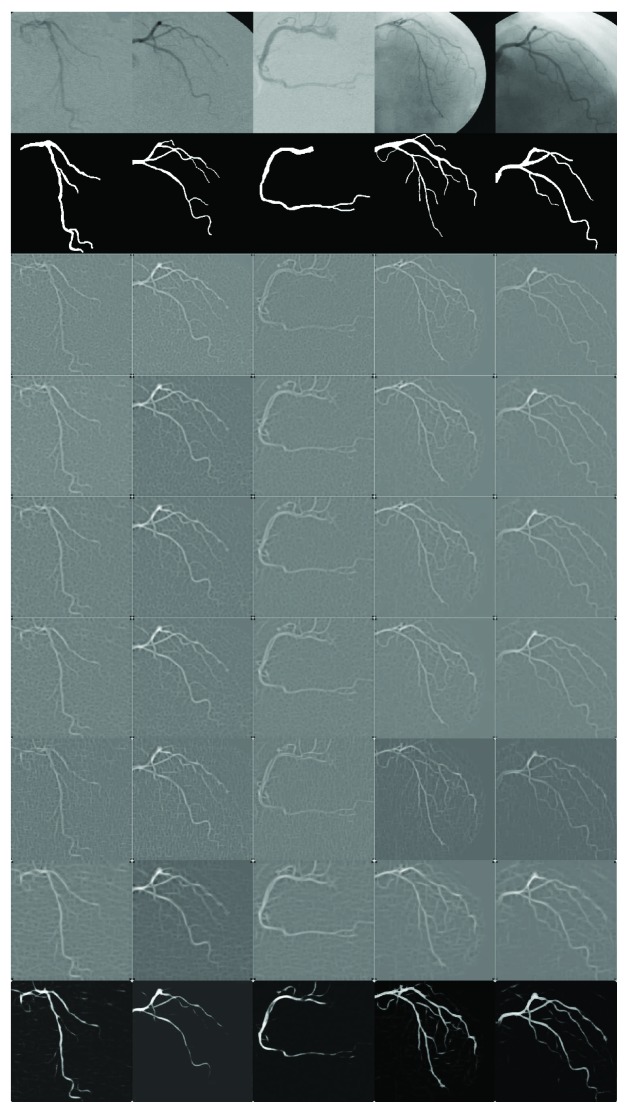
First row: subset of angiograms from the test set. Second row: ground truth of images. The remaining seven rows present the GMF response of the methods of GMF-Entropy [19], Kang et al. [18, 20, 21], Chaudhuri et al. [12], Cinsdikici and Aydin [16], Al-Rawi et al. [15, 22], GMF-Evol [23], and the proposed method (MGMF), respectively.

The vessel detection response of the six GMF-based methods and the proposed MGMF/AN(3-8) are presented in Figure 5 for a subset of five coronary angiograms from the testing set. The MGMF/ANN(3-8) responded with a greater visible contrast between the detected coronary artery and the background than the comparative GMF methods.

### 3.3. Results of Vessel Segmentation

The extraction of the coronary artery is completed through the classification of the MGMF response into vessel and nonvessel pixels. The classification of the detection response has been commonly carried out by a thresholding algorithm, which defines a limit value with the purpose of separating the vessel pixels from the background image. In order to take advantage of the high detection performance of the MGMF response, ten different thresholding methods from the state of the art were tested with the interest of defining the ideal coronary artery segmentation method.

Methods based on the entropy, such as the method of Kapur et al. [29] and the method of Pal and Pal [30], optimize the location of the value *t* which separates the histogram of the gray-scale image into two classes, with the objective of maximize the entropy of the two resulting classes. The moment-preserving method introduced by Tsai [31] assumes that the first three moments of the resulting binary image must be preserved. The threshold value *t* is defined as the location in the histogram of the gray-scale image which solves four predefined equations. The method of Rosenfeld and De la Torre [32] referred as the histogram concavity algorithm, which works with the convex hull of the histogram of the gray-scale image. The threshold value *t* is defined as the location of the local maxima of the difference between the convex hull and the image histogram. The Rutherford-Appleton threshold selection (RATS) method [33] defines a maximum gradient image from the derivatives in the *x* and *y* directions of the input image. The threshold value *t* is defined as the division of the sum of every element of the dot product between the maximum gradient image and the input image, divided by the sum of every element of the maximum gradient image. In a probability distribution approach, the method of Otsu [34] proposes the selection of the threshold value *t* as the location in the histogram of the gray-scale image which maximizes the between-class variance of the resulting two classes. In a similar approach, Ridler and Calvard [35] model the two classes of the binary image using two Gaussian distributions. The resulting classes are separated by a threshold value *t*, defined as the average of the location parameters of the two Gaussian distributions, which are estimated through an iterative search. The method of White and Rohrer [36] classify each pixel using a local threshold value, which is defined as the expected intensity value of the corresponding neighborhood. Niblack [37] proposed the use of a similar neighborhood-defined threshold value, which also considers the local standard deviation. Finally, the method of Sauvola and Pietikäinen [38] defines a threshold value for each pixel according to the magnitude of the local standard deviation. This approach increases the threshold value for neighborhoods with low spread of the intensity and decreases the threshold value in neighborhoods with high standard deviation.

Table 3 presents the accuracy of the ten thresholding methods; the MGMF response of the testing set of angiograms was used as input. The comparative analysis shows that the thresholding method proposed by Otsu [34] produces the most accurate segmentation among the ten comparative methods; therefore, the method of Otsu [34] is used to binarize the MGMF response in further analysis.

The proposed MGMF/Otsu method for coronary artery segmentation was compared with seven vessel segmentation methods from the state of the art using the testing set of coronary angiograms.  Table 4 presents the comparative analysis of the vessel segmentation accuracy of the proposed MGMF/Otsu method and seven state-of-the-art methods. The experimental results of the comparative analysis show that the MGMF/Otsu outperforms the state-of-the-art methodologies in terms of the vessel extraction accuracy.

Finally, the execution time of the proposed MGMF/Otsu method and the seven state-of-the-art methods is shown in Table 5. The methods of Eiho and Qian [1] and Chanwimaluang et al. [13, 14] present the two lower execution times; however, those methods also produce low accurate segmentation of the coronary arteries from the testing set. The methods of Li et al. [9] and Wang et al. [8] were executed, respectively, in the third and fourth lower times, although those methods performed similarly and obtained an accuracy below to 94%. The proposed MGMF/Otsu method provides the best trade-off between accuracy and computational time, according to the experimental results, with an execution time of 1.73 seconds.

**Figure 6:**
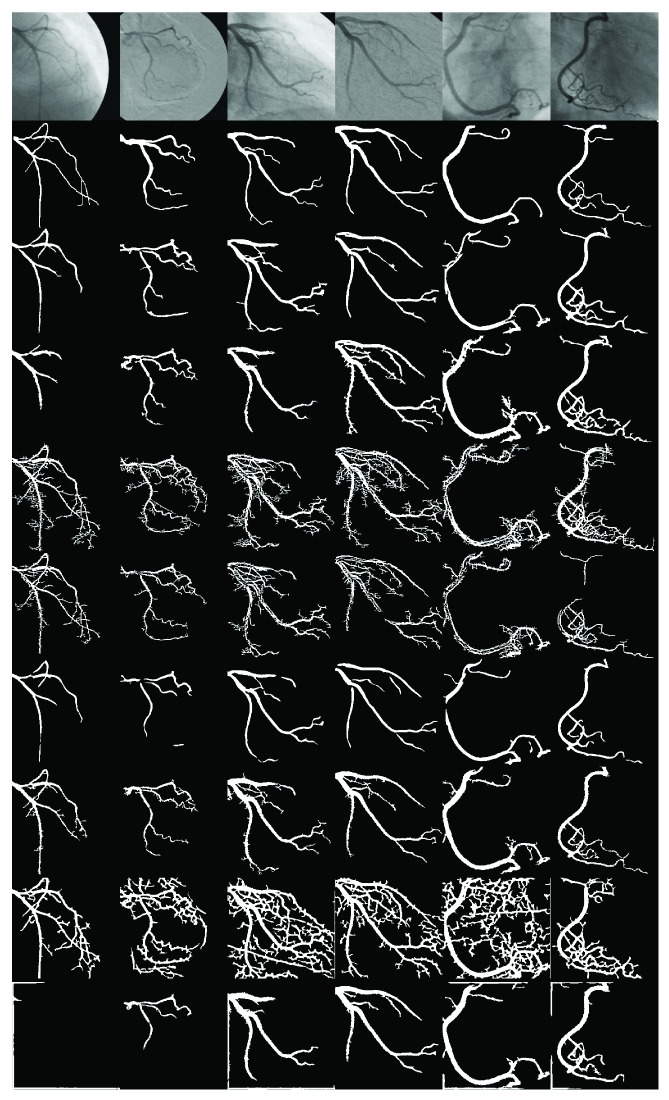
First row: subset of angiograms from the test set. Second row: ground truth of images. The remaining eight rows present the segmentation results of the proposed MGFM method, Kang et al. [21], Tsai et al. [10], Li et al. [9], Wang et al. [8], Eiho and Qian [1], Chanwimaluang et al. [13, 14], and Kang et al. [18], respectively.

Figure 6 shows the segmentation responses of the proposed MGMF/Otsu method and the seven state-of-the-art methods. The methods of Tsai et al. [10], Li et al. [9], and Chanwimaluang et al. [13, 14] present a high number of false positives, represented by the white pixels classified as vessels that are absent in the ground truth. Conversely, the methods of Kang et al. [21], Wang et al. [8], and Kang et al. [18] fail to extract thin vessels that are present in the ground truth, producing a high rate of false negative pixels. By visual examination of the Eiho and Qian [1] method responses, it noticed the presence of jagged edges, which reduce the segmentation accuracy. On the other hand, the segmentation responses of the proposed MGMF/Otsu method present smooth edges with an acceptable compromise of true-positive and false-negative pixels.

The comparative results of the performed experiments suggest that the proposed MGMF/Otsu method is robust for the coronary arteries detection and is capable of providing accurate segmentations from X-ray coronary angiograms, within a competitive computational time. The efficiency of the proposed MGMF/Otsu method encourages its usage to aid the decisions making in the medical practice.

## 4. Conclusion

In this paper, a novel method based on multiscale Gaussian-matched filters and artificial neural networks approach has been introduced. The statistical results show the robustness of the optimal architecture found for the ANN, which uses 3 neurons in the first hidden layer, and 8 neurons in the second. The optimal design reached a detection rate of 0.9357 in terms of the area (*A*_z_) under the ROC curve, using the training set of 50 angiograms. Further analysis showed that the proposed MGMF/ANN(3-8) method performs better than six GMF-based methods from the state of the art, presenting an *A*_z_ = 0.9362 using the testing set of 50 images. The segmentation of the MGMF/ANN(3-8) response carried out by the thresholding method of Otsu [34] has proven to be the most efficient from ten of the state-of-the-art segmentation techniques. According to the experimental results, the application of an artificial neural network of optimal architecture, over the responses of the multiscale Gaussian-matched filters, and the subsequent threshold of the response by the Otsu's method provide the most accurate segmentation of the coronary artery, with a correspondence of 0.9568, in a competitive execution time of 1.73 seconds.

## Figures and Tables

**Table 1:** Statistical analysis using 30 runs of the MGMF over the training set of X-ray angiograms.

Method	Maximum	Minimum	Mean	Std. dev.	Median
MGMF/ANN(3-8)	0.9357	0.9155	0.9203	0.002	0.9169

**Table 2:** Comparative analysis of vessel detection performance using the training and testing sets in terms of the area under the ROC curve.

	Area under ROC curve (*A*_z_)	
Vessel detection method	Training set	Testing set
GMF-Entropy [19]	0.8849	0.8812
Kang et al. [18, 20, 21]	0.8901	0.8852
Chaudhuri et al. [12]	0.9012	0.8963
Cinsdikici and Aydin [16]	0.9087	0.9002
Al-Rawi et al. [15, 22]	0.9104	0.9123
GMF-Evol [23]	0.9142	0.9171
Proposed method (MGMF)	0.9357	0.9362

**Table 3:** Comparative analysis of ten thresholding methods of the state of the art using the multiscale Gaussian response over the test set of X-ray angiograms.

Thresholding method	Accuracy
Otsu [34]	0.9568
Moments [31]	0.9561
Ridler and Calvard [35]	0.9560
RATS [33]	0.9533
Kapur et al. [29]	0.9514
Sauvola and Pietikäinen [38]	0.9290
Histogram concavity [32]	0.9254
Pal and Pal [30]	0.8982
Niblack [37]	0.8644
White and Rohrer [36]	0.8304

**Table 4:** Comparative analysis of the proposed method (MGMF) with respect to seven state-of-the-art vessel segmentation methods using the test set of 50 X-ray images.

Segmentation method	Accuracy
MGMF/Otsu	0.9568
Kang et al. [21]	0.9417
Tsai et al. [10]	0.9402
Li et al. [9]	0.9394
Wang et al. [8]	0.9386
Eiho and Qian [1]	0.9271
Chanwimaluang et al. [13, 14]	0.9150
Kang et al. [18]	0.8843

**Table 5:** Average execution time for the proposed method as compared with to the state-of-the-art segmentation methods using the test set of angiograms.

Segmentation method	Execution time (seconds)
MGMF/Otsu	1.73
Kang et al. [21]	2.51
Tsai et al. [10]	1.91
Li et al. [9]	1.52
Wang et al. [8]	1.63
Eiho and Qian [1]	1.05
Chanwimaluang et al. [13, 14]	1.24
Kang et al. [18]	2.01
